# Understanding Men’s Perceptions of Human Papillomavirus and Cervical Cancer Screening in Kampala, Uganda

**DOI:** 10.1200/JGO.17.00106

**Published:** 2018-02-07

**Authors:** Erin Moses, Heather N. Pedersen, Emily C. Wagner, Musa Sekikubo, Deborah M. Money, Gina S. Ogilvie, Sheona M. Mitchell-Foster

**Affiliations:** **Erin Moses**, **Emily C. Wagner**, **Deborah M. Money**, and **Gina S. Ogilvie**, Women’s Health Research Institute, BC Women’s Hospital + Health Centre; **Heather N. Pedersen**, University of British Columbia, Vancouver, British Columbia, Canada; and **Musa Sekikubo**, **Deborah M. Money**, **Gina S. Ogilvie**, and **Sheona M. Mitchell-Foster**, Makerere University, Kampala, Uganda.

## Abstract

**Purpose:**

This preliminary study explores Ugandan men’s knowledge and attitudes about human papillomavirus (HPV), cervical cancer, and screening.

**Methods:**

A local physician led an education session about cervical cancer for 62 men in Kisenyi, Kampala in Uganda. Trained nurse midwives administered surveys to assess knowledge and attitudes before and after the education session.

**Results:**

From the pre-education survey, only 24.6% of men had heard of HPV previously, and 59% of men had heard of cervical cancer. Posteducation, 54.5% of men believed only women could be infected with HPV and 32.7% of men believed antibiotics could cure HPV. Despite their limited knowledge, 98.2% of men stated they would support their partners to receive screening for cervical cancer, and 100% of men surveyed stated they would encourage their daughter to get the HPV vaccine if available.

**Conclusions:**

Knowledge of HPV and cervical cancer among Ugandan men is low. Even after targeted education, confusion remained about disease transmission and treatment. Ongoing education programs geared toward men and interventions to encourage spousal communication about reproductive health and shared decision making may improve awareness of cervical cancer prevention strategies.

## INTRODUCTION

Cervical cancer is one of the most common cancers among women globally, particularly in low- and middle-income countries (LMICs), which account for > 80% of new cases and 90% of all cervical cancer deaths.^1^ In these countries, including Uganda, the lack of population-level implementation of cervical cancer screening and human papillomavirus (HPV) vaccination has led to high rates of morbidity and mortality from this highly preventable disease. The understanding of HPV as a necessary cause for cervical cancer^2^ has created a paradigm shift from an oncological lens to one of a sexually transmitted infection. This has social and cultural implications that threaten the uptake of screening, particularly in low-resource settings where knowledge of HPV is low.^3^

Detection of high-risk strains of HPV DNA has emerged as a highly sensitive and effective tool for screening.^4^ Using this method, either a clinician or the woman herself can collect a cervical or vaginal specimen using a collection device, usually a swab, which is sent for laboratory testing. HPV self-collection, or self-sampling, is of particular interest in low-resource settings because it offers the opportunity to perform screening outside of the clinic setting. HPV testing is less invasive than traditional methods, such as cytology-based screening with a Papanicolaou smear test and visual inspection with acetic acid, and is more-cost effective than cytology.^5^ Published data indicate that HPV self-collection is highly acceptable among women living in LMICs compared with other screening methods.^6-8^ However, for successful implementation of new technologies, programs must consider the broader sociocultural factors within countries that could lead to unintended consequence or barriers. Previous research led by this team explored strategies for community engagement and education from the perspective of women and health care providers and identified that the lack of male involvement was a key barrier to cervical cancer screening.^9^

Several factors influence the success of cervical cancer screening programs in settings such as sub-Saharan Africa. Limited availability of screening, long distances to the clinic, poorly equipped health facilities, long wait times, embarrassment of receiving a pelvic examination, and lack of knowledge or risk perception for cervical cancer have all been established as major factors.^10,11^ More recently, there has been an increased interest in better understanding the role of male partners in cervical cancer screening attendance. In Uganda, gender plays a central role in health decision making.^11,12^ From assistance with child care to financial and emotional support, male partners can affect whether women receive reproductive health services.^11^ Issues including stigma, cultural taboos, and a general lack of knowledge have been described as barriers to supportive male engagement in women’s health care.^11-13^ The WHO has recently called for an increase in male involvement in the prevention of cervical cancer in LMICs.^14^ However, there are limited data on men’s knowledge of HPV and cervical cancer and their willingness to support their partners to be screened.^11,15,16^

Advances in Screening and Prevention in Reproductive Cancers (ASPIRE^17^) is an international women’s health initiative that uses innovations in technology to increase women’s access to reproductive health care.^6,18^ ASPIRE, established in 2006, has taken a pragmatic, implementation-focused approach to increasing access to cervical cancer screening in Kisenyi, a densely populated urban community in Kampala, Uganda. A key component of the ASPIRE model has been community mobilization and engagement before intervention to improve acceptability and uptake. With the goal to improve and scale up screening, we explored men’s knowledge and attitudes toward HPV and cervical cancer and intention to support partners to attend screening in a community where ASPIRE has conducted self-collection–based cervical cancer screening activities. Here we report on the results of a survey conducted before and after an education session with men and explore factors related to whether they had heard of cervical cancer before the education session.

## METHODS

This study was conducted as part of a randomized controlled trial comparing two cervical cancer screening tools, HPV self-collection and visual inspection with acetic acid, in Kisenyi.^7^ To better explore the role of partners in screening, the team conducted a separate study to explore men’s attitudes toward cervical cancer screening. In June 2015, outreach workers recruited men between the ages of 18 and 69 years through opportunistic recruitment in the area of the Kisenyi Health Centre and invited them to participate in an education session. Incentives were not offered to participants. The education session took place at the Kisenyi Health Center, an urban health center in Kampala, Uganda. As part of the program evaluation, participants completed a survey before and after the education session. A 2-hour presentation was run by the medical director of the Kisenyi Health Center and focused on HPV, cervical cancer, prevention methods, and screening options for women.

The survey tool (Data Supplement) was developed based on a comprehensive literature review of health education materials designed for LMICs, and a pre- and postintervention method was used for data collection. Both surveys, which were created in English and translated into Luganda, were piloted with a team of nurse midwives and outreach workers from the community. Trained nurse midwives administered the surveys orally, in either English or Luganda, the official languages of the region, to all men. Pre-education surveys included four sections of questions on demographics, knowledge, risk, and partner support levels; the post-education survey included two sections incorporating knowledge and partner support levels.

The pre-education questions recorded basic demographics, including age, education level, religion, income, and sexual health practices. The next section included three, yes/no/unsure questions on men’s knowledge of HPV and cervical cancer, and a risk section included two yes/no/unsure questions on men’s perceived risk of acquiring HPV and their partners’ risk of cervical cancer. The posteducation survey included nine yes/no/unsure questions about men’s knowledge about HPV and cervical cancer, including risk factors, screening, and treatment options, and two yes/no/unsure questions on men’s level of support for their partner to receive screening and for their daughters to receive the HPV vaccine if available.

Survey results were compiled and analyzed using SPSS (v24; Chicago, IL). Descriptive statistics were generated for results of all survey questions. In addition, a univariate analysis was conducted using Pearson’s χ^2^ test or Fisher’s exact test to determine the association of having heard of cervical cancer to factors of interest. The responses to having heard of cervical cancer were dichotomized, where a response of unsure was counted as a negative response. Unadjusted odds ratios (ORs) were calculated for all variables that reached significance of *P* > .05.

## RESULTS

In total, 63 men attended the education session, and all participants completed the pre-education survey. Thirty-two, or just over half, of the men were married (52.5%), and their mean number of children was 2.44 (interquartile range, 4.50). The median age of participants was 28 years (interquartile range, 16.00 years), 45 (73.8%) had completed some secondary education or higher, and most (n = 42; 68.9%) participants were currently employed (Table 1).

**Table 1:**
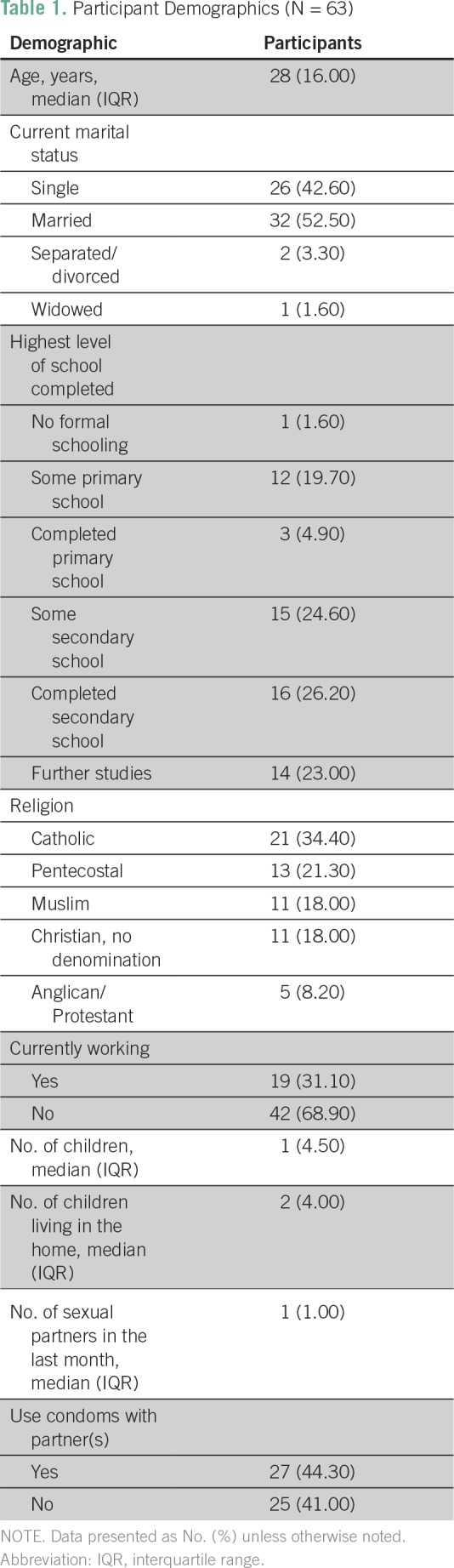
Participant Demographics (N = 63)

Before the education session, knowledge and awareness of cervical cancer was more than twice as high (n = 36; 59%) as knowledge of HPV (n = 15; 24.6%; Table 2). Of the men who had heard of HPV, only six (9.5%) believed it was transmitted through sexual intercourse. In total, 43 men (70%) indicated that their partner had never been screened for cervical cancer, and 57 (93%) would support their partner to be screened if screening was available in the community.

**Table 2:**
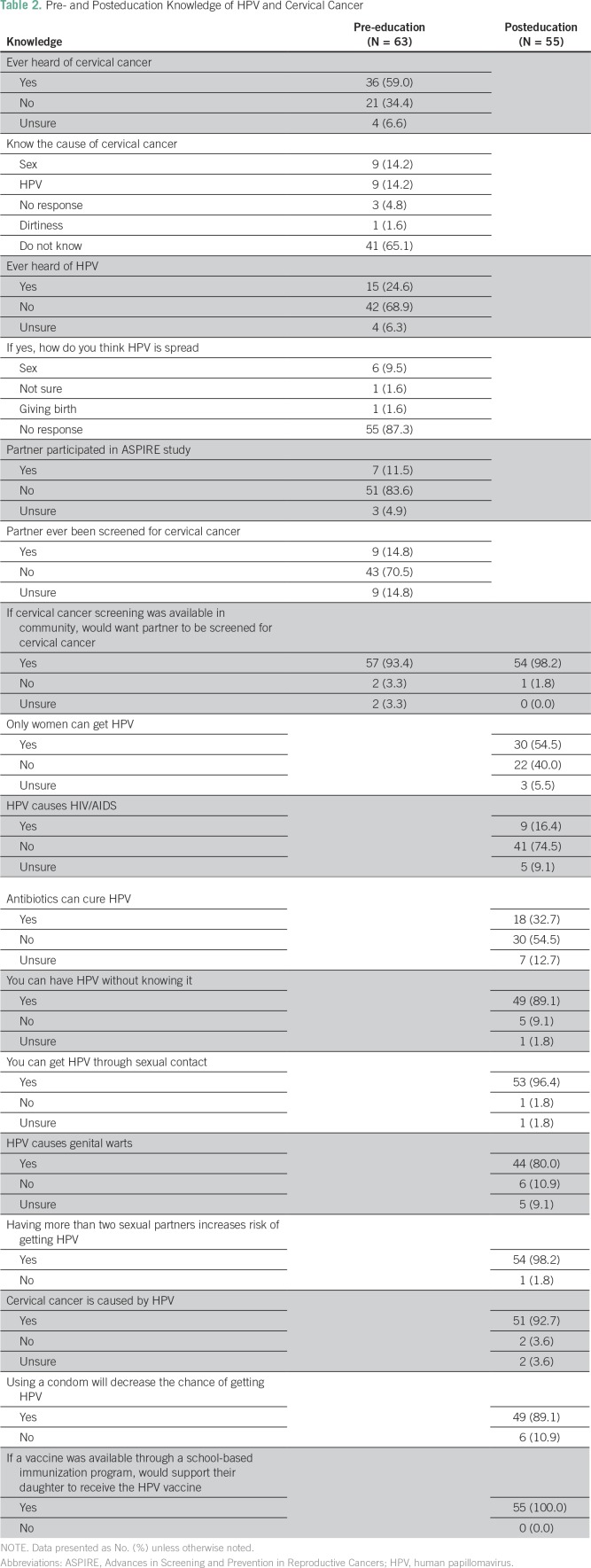
Pre- and Posteducation Knowledge of HPV and Cervical Cancer

Of the 62 men who completed the education session, 55 completed the posteducation survey (88.7%). Fifty-four men (98.2%) indicated they would want their partners to be screened for cervical cancer, and 55 (100%) would want their daughters to be vaccinated with the HPV vaccine if available. Forty-nine men (89.2%) understood that you could have HPV without knowing it, and 53 (96.4%) believed HPV was spread through sexual contact. However, some misconceptions about HPV remained after the education session was complete. Thirty men (54.5%) believed that only women could get HPV, nine (16.4%) believed that HPV causes HIV, and 18 (32.7%) believed that antibiotics could cure HPV.

From a univariate analysis comparing men who had previously heard of cervical cancer to those who had not, we found several variables of interest. Men who stated they had heard of cervical cancer on the pre-education survey were more likely to have fewer children in the home (*P* = .02), and had more sexual partners (*P* = .03) within the last month (Table 3). Men who had heard of cervical cancer were also more likely to have heard of HPV (*P* = .001); were less likely to believe, incorrectly, that antibiotics cure HPV (*P* = .02); and indicated they used condoms with their sexual partners (*P* = .02).

**Table 3:**
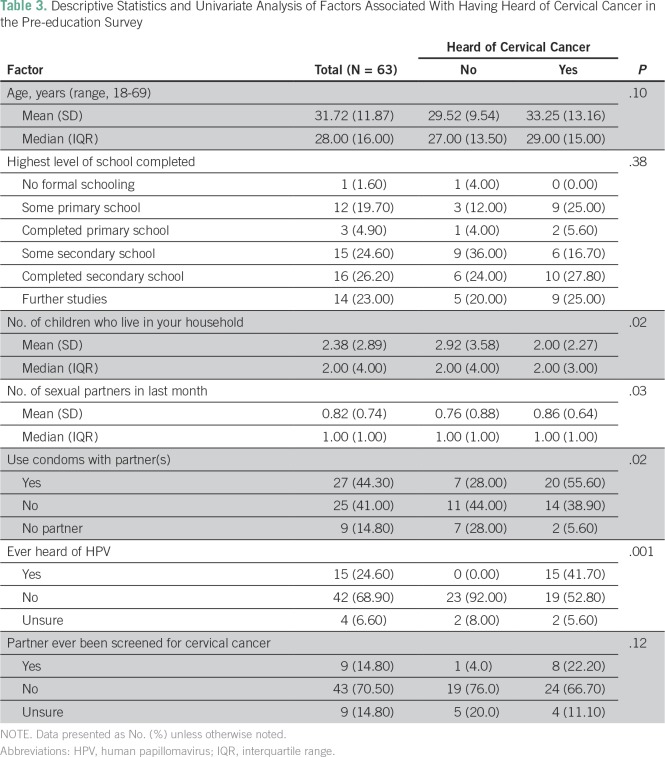
Descriptive Statistics and Univariate Analysis of Factors Associated With Having Heard of Cervical Cancer in the Pre-education Survey

An unadjusted OR of variables that reach a significance of *P* > .05 found that only one variable maintained statistical significance. Men who believed that antibiotics cure HPV were less likely to have heard of cervical cancer than those who did not (unadjusted OR, 0.18; CI, 0.05 to 0.63; Table 4).

**Table 4:**
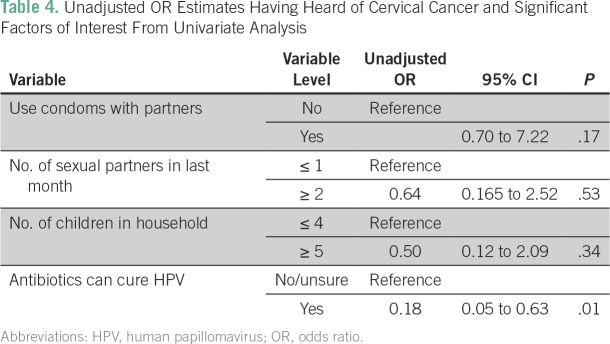
Unadjusted OR Estimates Having Heard of Cervical Cancer and Significant Factors of Interest From Univariate Analysis

## DISCUSSION

To our knowledge, this is the first study to provide preliminary evidence on Ugandan men’s knowledge and attitudes about HPV and cervical cancer and to assess their willingness to support their partners for cervical cancer screening. This research is important for the design and development of public health education strategies, because despite the emerging evidence of the role of partners in influencing screening, there are limited data on men’s desire and ability to influence women’s reproductive health. Importantly, the WHO has called for more male involvement in cervical cancer screening globally.^14^

Low knowledge of HPV and cervical cancer observed in this study is in line with other published data from LMICs, such as Kenya and Ghana.^15,16^ Previous studies of the impact of education on male involvement in antenatal and HIV care demonstrated that men’s desire for reproductive education is a critical component to sustainable positive health outcomes.^19^ In prior ASPIRE research on community engagement and education leading up to a cervical cancer clinical trial, women in the Kisenyi district indicated male involvement was central to the success of any cervical cancer screening intervention.^9^ In this group of men, even after the education session, some were still unsure of the risk factors for cervical cancer, and there remained confusion around how HPV transmission and treatment occurred. These findings highlight that a one-time didactic education session may not be sufficient for knowledge translation and the need for ongoing culturally appropriate education programs for both men and women about cervical cancer and HPV.

We found almost all men who participated were willing to support their partners to attend screening after the education session, and many asked additional questions about HPV transmission and whether it was necessary for men to get screened (E. Moses, personal communication, November 2015). Research has outlined that communication between spouses and shared decision making on aspects of reproductive health can improve family planning use,^20,21^ which could be relevant to cervical cancer screening. Future studies should explore male partners’ perceived barriers to their involvement in their partners’ screening as well as the impact of ongoing education on their partners’ reproductive health. It was interesting to note that 100% of men said that they would want their daughters vaccinated with the HPV vaccine if available. Previously published data suggest that cultural beliefs and limited education were a barrier to HPV vaccination in some LMICs but that these can be mitigated through community sensitization and education.^22^ Our study sample had the opportunity to learn and ask questions about HPV before responding to the vaccine question; therefore, they may better represent the acceptability after education and not in a naïve population

Several limitations could have influenced this study. Female midwives interviewed men; therefore, social desirability bias could have affected men indicating they would support their partners to be screened. To limit this, men were instructed to fill out their own responses, which were not monitored by the midwives. Furthermore, a male physician led the education session and question period, because it was believed men may feel more comfortable engaging in discussion with a male facilitator. This study was conducted in parallel to a cervical cancer screening trial; seven men (11.5%) had said they were partners of trial participants, and another three men (4.9%) were unsure. It is possible our study population is more supportive of screening, or more knowledgeable, than the general population. Further analysis showed that men who had partners who had previously participated in the study were more likely to have partners who had been screened previously, had more children and children living in the household, and were older. It is also likely that men’s report of whether their partner had ever been screened for cervical cancer could be inaccurate, as a result of the previously discussed barriers to male partner engagement in reproductive health. Cervical cancer screening rates in Uganda are sporadic and have been reported to be between 4.8% and 30%^23^; therefore, it is difficult to determine whether the 14.8% reported for men’s partners who had been screened ever (Table 2) is comparable. Because of the sample size and homogeneity of responses, we were not able to explore the predictors of men’s willingness to support screening.

In conclusion, cervical cancer is a leading cause of death for women in countries such as Uganda, and a lack of partner support is a barrier to screening. Men’s knowledge of cervical cancer and HPV remain low in many LMICs; however, men in this preliminary study expressed a high level of interest in reproductive health education and the desire to support their partner to be screened for cervical cancer. The posteducation survey indicated some improvements in knowledge after participating in a community education session. However, confusion remained in some areas, which highlights the need for ongoing education programs geared toward men. Findings from this study provide some evidence of the need for enhanced methods to engage and educate men to encourage men’s involvement in reproductive screening programs.
